# Intra-Abdominal Hemorrhage following Cardiopulmonary Resuscitation: A Report of Two Cases

**DOI:** 10.1155/2018/5243105

**Published:** 2018-06-04

**Authors:** Christos Koutserimpas, Argyrios Ioannidis, Petros Siaperas, Andreas Skarpas, Andreas Tellos, Georgios Velimezis, Ioannis Karanikas

**Affiliations:** 2nd Surgical Department, Sismanoglio General Hospital of Athens, Greece

## Abstract

Cardiopulmonary resuscitation (CPR) represents an emergency procedure, consisting of chest compressions and artificial ventilation. Two rare cases of intra-abdominal bleeding following cardiac compressions are reported. The first case was a 29-year-old female with massive pulmonary embolism (PE). Following CPR due to cardiac arrest, she showed signs of intra-abdominal bleeding. A liver laceration was found and sutured. The patient passed away, due to massive PE. The second patient was a 62-year-old female, suffering from cardiac arrest due to drowning at sea. CPR was performed in situ. At presentation to the emergency department she showed signs of intra-abdominal bleeding. The origin of the hemorrhage was found to be vessels of the lesser curvature of the stomach, which were ligated. Regarding the first patient PE has already been described as a cause for liver lacerations in CPR due to stasis and liver enlargement. The second case is the first report of gastric vessel injury without gastric rupture/laceration and pneumoperitoneum. Complications of CPR should not represent a drawback to performing cardiac compressions. Parenchymatic injuries have been related to inappropriate technique of chest compressions during basic life support. Therefore, it is of utmost importance for the providers to refresh their knowledge of performing CPR.

## 1. Introduction

Cardiopulmonary resuscitation (CPR) represents an emergency procedure, consisting of chest compressions and artificial ventilation. It is a procedure, performed in cases of cardiac arrest, aiming to manually preserve circulatory flow and oxygenation. CPR, for adults, includes chest compressions between 5 cm and 6 cm depth at a rate of about 120 per minute [1].

The most common complications arising from chest compressions include rib and sternal fractures [2]. Intra-abdominal bleeding is a rare complication of chest compressions. In the present article two cases of intra-abdominal hemorrhage following CPR are reported.

## 2. Case 1

A previously healthy 29-year-old female was being transferred to the “Sismanoglio” General Hospital of Athens, Greece, due to pulmonary embolism (PE) 24 hours following a caesarian section. She had already received a bolus of intravenous 5000 E Heparin. The patient was hemodynamically unstable (blood pressure= 90/40 mmHg, heart rate= 140 beats/min, saturation= 85%, and breathing rate= 30 breaths/min) and afebrile (37 C). During the transportation, she suddenly went into cardiopulmonary arrest with pulseless electrical activity. CPR was immediately instituted according to advanced life support guidelines. Manual chest compressions and artificial ventilation were performed. The CPR was effective and the patient was then transferred to the Intensive Care Unit (ICU). About 3 hours later, she continued being hemodynamically unstable, her hematocrit had dropped 10 points (from 33% at ICU admission to 23% at that point), and she had a distended abdomen. She was transfused with two blood units and an emergency Diagnostic Peritoneal Lavage (DPL) was positive for intra-abdominal bleeding. At that point she was urgently taken into the operating theatre. An exploratory laparotomy was decided upon. About two liters of blood was removed from the abdomen. The intraoperative finding was an approximately 5 cm liver laceration at the left lobe, near the attachment point of the left coronary ligament. Liver suturing was performed and the intra-abdominal bleeding was successfully controlled. Unfortunately the patient passed away 3 hours after the operation. Cause of death was the massive pulmonary embolism.

## 3. Case 2

A previously healthy 62-year-old female was transferred with an ambulance to the emergency department due to drowning at the sea. CPR had been instituted in situ by an experienced lifeguard. At presentation the patient was hemodynamically unstable (blood pressure= 100/55 mmHg, heart rate= 135 beats/min, and breaths= 25 per min) and afebrile (36.7 C). Her laboratory investigation showed hematocrit (Hct)= 23% and hemoglobin (Hgb)= 7.3 g/dL. Fluid resuscitation was performed and she was also transfused with 2 blood units, but she continued being hemodynamically unstable. After the transfusion she had Hct= 19% and Hgb= 6.4 g/dL. The abdominal ultrasonography revealed free intra-abdominal fluid. Bedsides, chest X-ray was negative for tympanic abdomen or pneumoperitoneum. At that point an exploratory laparotomy was decided upon. The site of bleeding was found to be vessels originating from the lesser curvature of the stomach, which were successfully ligated. The patient had an uneventful recovery and was discharged on the 12th postoperative day. Eight years after the event, she continues her everyday activities, without any limitations.

## 4. Discussion

Rib and sternal fractures represent the main complications following CPR [2]. Life-threatening complications have also been reported. These include heart contusion, hemopericardium, pneumothorax, hemothorax, lung contusions, and intra-abdominal injuries, including liver and spleen lacerations [3, 4]. There have also been described cases of traumatic pancreatitis and colon perforation [5, 6].

The first patient went into cardiac arrest during transportation. The patient was already diagnosed with PE. The most likely cause was the massive pulmonary embolism, which is consistent with the tachypnea and the hemodynamic instability. Liver injury associated with chest compressions is the most commonly observed parenchymatic injury, occurring at a rate of 0.6%–2.9% [3, 4, 7]. Meron et al. found it in 0.6% (15/2558), Kralj et al. in 0.6% (13/2148), Patterson et al. in 0.8% (3/377), and Krischer et al. in 2.9% (20/705) of the resuscitated cases [2, 3, 7, 8]. However, in the latter series 46.5% of the 2187 cardiac arrests were due to nonnatural causes and 63.5% of the patients had been resuscitated with the use of mechanical cardiocompression devices.

Massive PE is associated with acute right ventricular failure, since it increases the pulmonary artery pressure. Additionally, stasis from the right ventricular failure can also lead to liver-failure and enlargement [9]. Although this postulation is appealing, there are not any studies supporting it, since it is relatively rare. However, there are few cases reporting liver injury following cardiac compressions in patients with PE [4, 10]. PE compromises cardiac function, leading to parenchymal organ stasis. This seems to be a possible risk factor for parenchymatic injury and internal hemorrhage caused by external forces (chest compressions) [9]. Furthermore, the use of Heparin in the present case should be taken into account, since it could also create hemorrhagic predisposition.

The second patient suffered from cardiac arrest after drowning at the sea. CPR was instituted in situ. During surgery the origin of the intra-abdominal hemorrhage was found to be vessels originating from the lesser curvature of the stomach. Gastric rupture following CPR seems to be extremely rare. However, gastric dilatation has been reported at autopsies. Krischer et al. observed 29.1% gastric dilation in autopsies but found gastric rupture in only 1/705 autopsies and Kralj et al. in only 1/2148 cases [2, 8]. Spoormans et al. after reviewing 67 cases of gastric perforation following CPR reported that, despite the common occurrence of gastric distension, massive gastric distension leading to gastric perforation secondary to CPR has been rarely reported [11, 12]. Bedsides, chest X-ray of the present patient did not reveal pneumoperitoneum. The absence of these signs implicates that the gastric vessel injury should be ascribed solely to the CPR and not to improper ventilation which could have contributed to gastric or gastric vessel injury. To the best of our knowledge, CPR-related gastric vessel injury without gastric rupture or laceration and pneumoperitoneum has not been previously reported.

Both reported cases were females. Hallevuo et al. reported slightly more rib (31% versus 20%) and sternal (11% versus 9%) fractures in males. It is noteworthy that in that study resuscitation attempts on males lasted longer than on females [12]. On the other hand, Kralj et al. found liver ruptures in 2 males and 11 females, CPR-related rib fracture rates of 77% in males and 85% in females, and sternum fracture rates of 59% in males and 79% in females [2]. Additionally, Meron et al. observed liver ruptures in 0.6% of male and 0.5% of female patients [3]. It seems that the influence of gender remains unclear.

It is of note that although the first patient passed away, the cause of death was not the intra-abdominal bleeding. Therefore, both life-threatening complications were successfully managed.

During the last two decades it has been reported that the faster and deeper chest compressions improve the survival rate [6]. This led to changes in the CPR guidelines issued in 2010 and 2015 by the American Heart Association and the European Resuscitation Council. Increase in both the number of compressions per minute and compression depth was recommended, in comparison to the guidelines of 2005 [6, 12]. Iatrogenic abdominal parenchymatic injuries could be associated with deeper chest compressions. There are reports also in cases of automatic mechanical chest compression that support this hypothesis [12, 13]. Kralj et al. reported 0.85% (3/353) CPR-related iatrogenic injury rate in the period 2004–2005, increase of these injury rate to 1.31% (14/1072) in the period 2006–2010, and further increase to 1.85% (14/723) in the period 2011–2013 [2]. On the other hand, Meron et al. reported on liver injuries in 15/2558 cardiac arrest victims resuscitated in the period 1991–2005 and Krischer et al. reported liver ruptures in 15 (2.1%) and liver lacerations in 5 (0.8%), spleen ruptures in 2 (0.3%), and gastric rupture in 1 (0.1%) of 705 patients who were autopsied in the period 1977–1978, which is long before the implementation of ERC guidelines 2010 [3, 7]. Therefore, data from the literature do not support the hypothesis that life-threatening complications have mainly been associated with the change of guideline into faster and deeper ones.

In conclusion, CPR is an emergency procedure that has to be performed in cases of cardiac arrest. Possible complications should, of course, not deter the providers from performing chest compressions. It has been suggested that parenchymatic injuries are related to an inappropriate technique of chest compressions during basic life support [7, 14]. They have been associated not only with improper hand placement, but also with excessive pressure during compressions, leading to severe crush injury of viscera. However, such complications have been observed even in cases where compressions were correctly performed [3].

It is of utmost importance for the providers to refresh the knowledge of performing CPR. Compressions of the right depth together with the correct placement of the hands by the CPR provider could minimize the complication rate. CPR represents the only and last hope of survival in cases of cardiac arrest. The risk of complications does not overshadow the benefits of a successful CPR.
